# Mori Ramulus Suppresses Hydrogen Peroxide-Induced Oxidative Damage in Murine Myoblast C2C12 Cells through Activation of AMPK

**DOI:** 10.3390/ijms222111729

**Published:** 2021-10-29

**Authors:** Cheol Park, Seon Yeong Ji, Hyesook Lee, Sung Hyun Choi, Chan-Young Kwon, So Young Kim, Eun Tag Lee, Sung Tae Choo, Gi-Young Kim, Yung Hyun Choi, Mi Ryeo Kim

**Affiliations:** 1 Division of Basic Sciences, College of Liberal Studies, Dong-Eui University, Busan 47340, Korea; parkch@deu.ac.kr; 2 Department of Biochemistry, College of Korean Medicine, Dong-Eui University, Busan 47227, Korea; 14602@deu.ac.kr (S.Y.J.); 14769@deu.ac.kr (H.L.); 3 Anti-Aging Research Center, Dong-Eui University, Busan 47340, Korea; 4 Department of System Management, Korea Lift College, Geochang 50141, Korea; choisunghyun@klc.ac.kr; 5 Department of Oriental Neuropsychiatry, College of Korean Medicine, Dong-Eui University, Busan 47340, Korea; beanalogue@deu.ac.kr; 6 Department of Pharmacology, College of Korean Medicine, Daegu Haany University, Daegu 42158, Korea; un9900@dhu.ac.kr; 7 Agricultural Corporation, Ebiche Co., Ltd., Yeongcheon 38819, Korea; etlee206@naver.com (E.T.L.); choo1656@hanmail.net (S.T.C.); 8 Department of Marine Life Science, School of Marine Biomedical Sciences, Jeju National University, Jeju 63243, Korea; immunkim@jejunu.ac.kr

**Keywords:** Mori Ramulus, myoblast, ROS, apoptosis, AMPK, muscle atrophy

## Abstract

Mori Ramulus, the dried twigs of *Morus alba* L., has been attracting attention for its potent antioxidant activity, but its role in muscle cells has not yet been elucidated. The purpose of this study was to evaluate the protective effect of aqueous extracts of Mori Ramulus (AEMR) against oxidative stress caused by hydrogen peroxide (H_2_O_2_) in C2C12 mouse myoblasts, and in dexamethasone (DEX)-induced muscle atrophied models. Our results showed that AEMR rescued H_2_O_2_-induced cell viability loss and the collapse of the mitochondria membrane potential. AEMR was also able to activate AMP-activated protein kinase (AMPK) in H_2_O_2_-treated C2C12 cells, whereas compound C, a pharmacological inhibitor of AMPK, blocked the protective effects of AEMR. In addition, H_2_O_2_-triggered DNA damage was markedly attenuated in the presence of AEMR, which was associated with the inhibition of reactive oxygen species (ROS) generation. Further studies showed that AEMR inhibited cytochrome *c* release from mitochondria into the cytoplasm, and Bcl-2 suppression and Bax activation induced by H_2_O_2_. Furthermore, AEMR diminished H_2_O_2_-induced activation of caspase-3, which was associated with the ability of AEMR to block the degradation of poly (ADP-ribose) polymerase, thereby attenuating H_2_O_2_-induced apoptosis. However, compound C greatly abolished the protective effect of AEMR against H_2_O_2_-induced C2C12 cell apoptosis, including the restoration of mitochondrial dysfunction. Taken together, these results demonstrate that AEMR could protect C2C12 myoblasts from oxidative damage by maintaining mitochondrial function while eliminating ROS, at least with activation of the AMPK signaling pathway. In addition, oral administration of AEMR alleviated gastrocnemius and soleus muscle loss in DEX-induced muscle atrophied rats. Our findings support that AEMR might be a promising therapeutic candidate for treating oxidative stress-mediated myoblast injury and muscle atrophy.

## 1. Introduction

Excessive production of reactive oxidative species (ROS) due to redox imbalance and impaired antioxidant defense systems has been shown to cause oxidative damage in most organs, including muscles [1,2,3]. In particular, in skeletal muscle cells, which consume more oxygen than other cells, mitochondria are overactivated according to contractile activity, resulting in increased ROS production [3,4,5]. Oxidative stress following increased ROS accumulation also promotes proteolysis, leading to muscle atrophy, and ROS serve as important mediators of multiple signaling pathways regulating this process [6,7]. In addition, overproduction of ROS results in cell death, including apoptosis and necrosis, following oxidative damage to intracellular macromolecules such as nucleic acids, lipids, and proteins [8,9]. Moreover, overproduced ROS can reduce mitochondrial membrane potential (MMP, *Δψm*), one of the hallmarks of mitochondrial dysfunction. Subsequently, apoptogenic factors such as cytochrome *c*, which exist between the inner and outer membranes of mitochondria, are released into the cytoplasm, and the caspase cascade is activated to cause apoptosis [10,11]. Therefore, the application of antioxidants to relieve ROS-induced oxidative stress, thereby controlling oxidative stress and regulating the redox system, is considered a promising approach for preserving muscle function.

Many herbs that have been used in traditional medicine for centuries are a source of drug discovery for the treatment and management of various diseases, and have the advantage of having fewer side effects compared to synthetic drugs [12,13]. *Morus alba* L. (Mulberry, Chilcompton, UK), which is widely used as a traditional medicine and belongs to the Mulberry family, is a perennial woody tree native to China [11,14]. Many previous studies have revealed that *M. alba* has promising pharmacological potentials, including anti-inflammatory, anti-diabetic, immunomodulatory, anti-obesity, anti-cancer, and liver and kidney protection [11,15,16]. This pharmacological activity in muscle cells is thought to correlate at least with redox signaling pathways involved in the regulation of energy metabolism. For example, extracts of *Mori Folium*, the dried leaves of *M. alba*, could improve insulin sensitivity and hyperglycemia by inhibiting gluconeogenesis in the liver of skeletal muscle, and that activation of AMP-activated protein kinase (AMPK), an energy sensor, was involved in this process [17,18]. Recently, Meng et al. [19] also reported that flavonoids isolated from *Mori Folium* enhanced mitochondrial function of skeletal muscle through activation of AMPK in db/db mice and L6 myoblasts. These results highly support the results of other studies, suggesting that AMPK is a key regulator in the antioxidant activity of *Mori Folium* [20,21]. However, it is not well known whether the antioxidant activity of *Mori Ramulus* is exerted in muscle cells, and studies related to the AMPK signaling have also not been conducted to date. Therefore, in this study, we evaluated the protective effect of aqueous extracts of *Mori Ramulus* (AEMR) against hydrogen peroxide (H_2_O_2_)-caused oxidative stress in C2C12 mouse myoblasts, and explored relevant signaling pathways to elucidate the potential of AEMR as an antioxidant. The therapeutic efficacy of calcitriol on skeletal muscle atrophy was also studied.

## 2. Results

### 2.1. AEMR Protected C2C12 Cells from H_2_O_2_-Induced Cell Viability Loss

**Figure 1:**
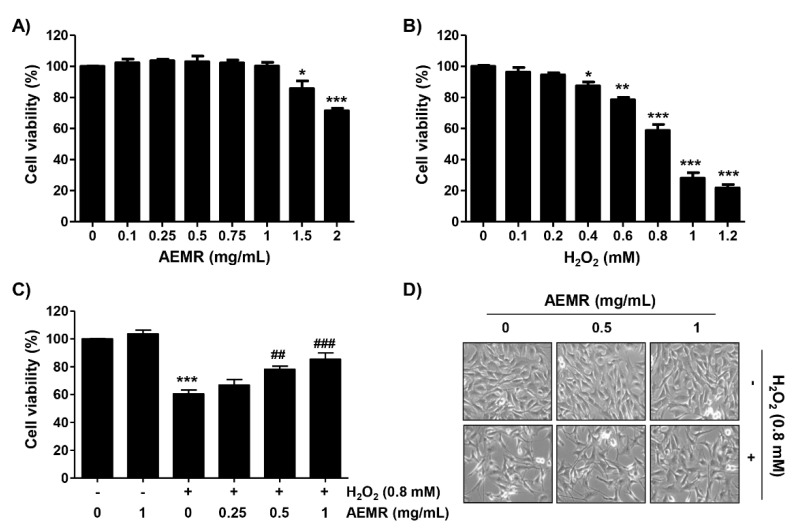
Effect of aqueous extracts of *Mori Ramulus* (AEMR) on hydrogen peroxide (H_2_O_2_)-induced growth inhibition in C2C12 cells. Cells were treated with different concentrations of AEMR or H_2_O_2_ for 24 h (**A**,**B**) or pretreated with or without the indicated concentrations of AEMR for 1 h prior to exposure to 0.8 mM H_2_O_2_ for 24 h (**C**,**D**). (**A**–**C**) Cell viability was evaluated using a 3-(4,5-dimethylthiazol-2-yl)-2,5-diphenyltetrazolium bromide (MTT) assay. Each point is the mean ± SD of independent triplicate experiments (* *p* < 0.05, ** *p* < 0.01 and *** *p* < 0.001 vs. control cells; ^##^ *p* < 0.01 and ^###^ *p* < 0.001 vs. H_2_O_2_-treated cells). (**D**) Morphological changes of H_2_O_2_-treated cells in the presence or absence of AEMR were observed by a phase-contrast microscope.

C2C12 cells were incubated with various concentrations of AEMR or H_2_O_2_ for 24 h, and the cell viability was measured using the 3-(4,5-dimethylthiazol-2-yl)-2,5-diphenyltetrazolium bromide (MTT) assay. As shown in Figure 1A, no cytotoxic effect was observed upon AEMR treatment up to 1 mg/mL concentration. Compared with the control group, H_2_O_2_ caused significant cytotoxicity in a concentration-dependent manner, starting at 0.4 mM, and cells treated with 0.8 mM H_2_O_2_ showed a survival rate of approximately 60% (Figure 1B). Therefore, the treatment concentration of H_2_O_2_ to induce oxidative stress was selected to be 0.8 mM, and the highest concentration of AEMR to investigate its protective effect was set to be 1 mg/mL without cytotoxicity. To investigate the protective effect of AEMR on H_2_O_2_-induced cytotoxicity, cells were pretreated with AEMR at 1 mg/mL or less for 1 h, and then treated with 0.8 mM H_2_O_2_ for 24 h. The result showed that pretreatment with AEMR significantly prevented the inhibition of cell survival by H_2_O_2_ in a concentration-dependent manner (Figure 1C). In addition, Figure 1D shows that H_2_O_2_ stimulation significantly induced morphological changes, including extensive cytosolic vacuolization and the presence of irregular cell-membrane buds, which were effectively attenuated by AEMR pre-treatment.

### 2.2. AEMR Prevented H_2_O_2_-Induced Mitochondrial Dysfunction in C2C12 Cells

**Figure 2:**
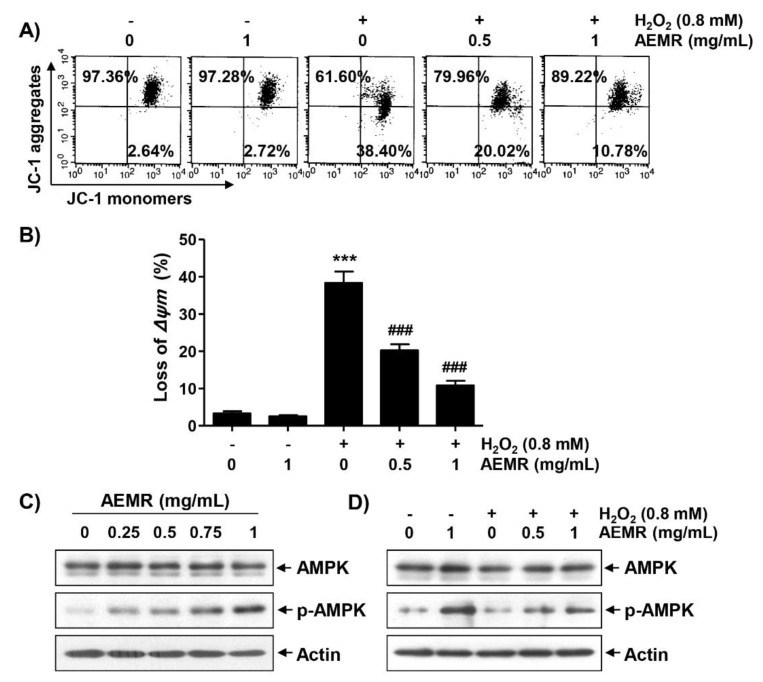
Attenuation of H_2_O_2_-induced mitochondrial dysfunction and activation of AMP-activated protein kinase (AMPK) by AEMR in C2C12 cells. Cells were treated with the indicated concentrations AEMR or 0.8 mM H_2_O_2_ for 24 h, or pretreated with AEMR for 1 h prior to exposure to H_2_O_2_ for 24 h. (**A**,**B**) Mitochondrial membrane potential (MMP) was assessed by flow cytometry after 5,5′6,6′-tetrachloro-1,1′,3,3′-tetraethyl-imidacarbocyanine iodide (JC-1) staining. (**A**) Representative profiles of flow cytometry analysis are shown. (**B**) Ratios of JC-1 aggregates to monomers are presented as mean ± SD of triplicate independent experiments (*** *p* < 0.001 vs. control cells; ^###^ *p* < 0.001 vs. H_2_O_2_-treated cells). (**C**,**D**) Expression of AMPK and p-AMPK was measured by western blot analysis. Anti-actin immunoblotting revealed relative amounts of protein in each lane.

Loss of MMP due to mitochondrial dysfunction is a hallmark of apoptosis triggered by H_2_O_2_ [10,22], and AMPK is known to be an important regulator of mitochondrial biosynthesis in skeletal muscle [4,23]. Therefore, we investigated whether AEMR could prevent H_2_O_2_-induced MMP loss, and regulate AMPK activation. As shown in Figure 2A,B, treatment with AEMR alone had no effect on MMP: the loss of MMP was significantly increased in cells treated with H_2_O_2_. However, the decline of MMP in H_2_O_2_-treated cells was significantly prevented in the presence of AEMR. In addition, although there was no change in the expression of the total amount of AMPK protein, AEMR markedly induced the phosphorylation of AMPK (p-AMPK), the active form of AMPK, in a concentration-dependent manner (Figure 2C). Additionally, the level of p-AMPK, which remained unchanged in H_2_O_2_ treatment alone, increased even in the presence of AEMR (Figure 2D), indicating that AEMR was able to stimulate AMPK activation in H_2_O_2_-treated C2C12 cells.

### 2.3. Inhibition of the AMPK Signaling Pathway Counteracted the Protective Effect of AEMR against the Cytotoxicity of H_2_O_2_ in C2C12 Cells

**Figure 3:**
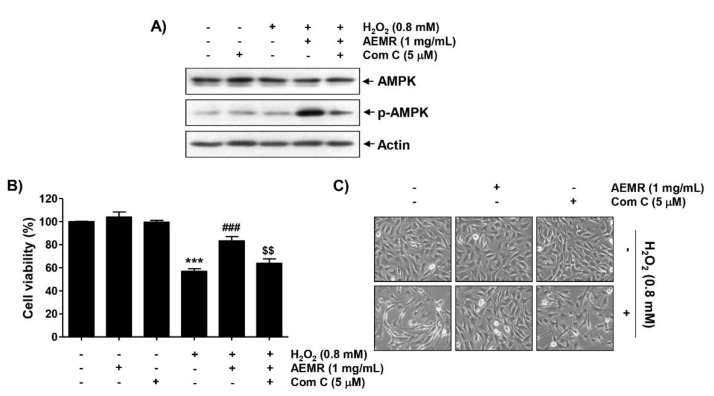
Effects of AMPK inhibitor, compound C, on AEMR-mediated protection of growth inhibition by H_2_O_2_ in C2C12 cells. Cells were pretreated with 5 μM compound C (Com C) with or without 1 mg/mL AEMR for 1 h, and then subjected to treatment with 0.8 mM H_2_O_2_ for 24 h. (**A**) Expression of AMPK and p-AMPK was measured by western blot analysis. Anti-actin immunoblotting revealed relative amounts of protein in each lane. (**B**) Cell viability was evaluated using an MTT assay. Data are presented as mean ± SD from triplicate experiments (*** *p* < 0.001 vs. control cells; ^###^ *p* < 0.001 vs. H_2_O_2_-treated cells; ^$$^ *p* < 0.001 vs. H_2_O_2_ and AEMR-treated cells). (**C**) Morphological changes were observed with a phase-contrast microscope.

To determine whether AMPK signaling was required for the protective effect of AEMR against H_2_O_2_-mediated cytotoxicity, we used the AMPK inhibitor compound C. Immunoblotting results revealed that compound C pretreatment greatly reversed the AEMR-induced upregulation of AMPK phosphorylation in H_2_O_2_-treated C2C12 cells (Figure 3A). Moreover, the protective effect of AEMR against growth inhibition and morphological changes by H_2_O_2_ was completely abrogated by compound C (Figure 3B,C), supporting the notion that AEMR protected C2C12 cells from oxidative stress-induced damage by activating AMPK.

### 2.4. AEMR Suppressed H_2_O_2_-Induced ROS Generation and DNA Damage in C2C12 Cells in an AMPK-Dependent Manner

**Figure 4:**
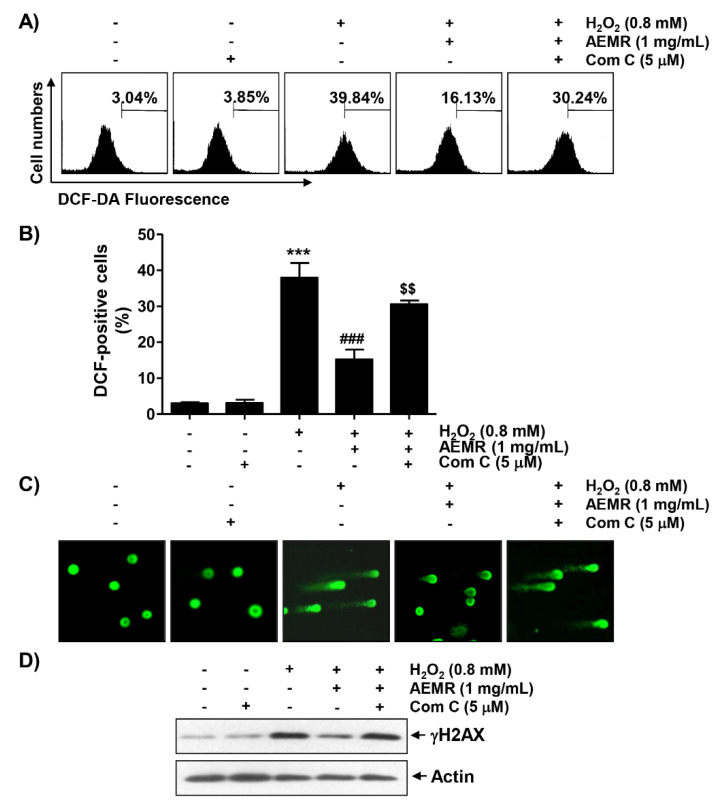
Effects of AMPK inhibitor on suppression of H_2_O_2_-induced ROS generation by AEMR in C2C12 cells. Cells were pretreated with 5 μM compound C with or without 1 mg/mL AEMR for 1 h, and then subjected to treatment with 0.8 mM H_2_O_2_ for 1 h (**A**,**B**) or 24 h (**C**,**D**). (**A**) Intracellular ROS levels were determined by flow cytometry after staining with 2′7′-di-chlorodihydrofluorescein diacetate (DCF-DA). (**B**) Ratios of DCF-positive cells were statistically quantified. Results are expressed as mean ± SD of three independent experiments (*** *p* < 0.001 vs. control cells; ^###^ *p* < 0.001 vs. H_2_O_2_-treated cells; ^$$^ *p* < 0.01 vs. H_2_O_2_ and AEMR-treated cells). (**C**) DNA damage was detected by a comet assay. Representative images are shown. (**D**) For western blot analysis, extracted proteins were subjected to sodium-dodecyl sulfate (SDS)-polyacrylamide gel electrophoresis and then transferred to polyvinylidene fluoride (PVDF) membranes. Membranes containing proteins were probed with indicated antibodies. Anti-actin immunoblotting revealed relative amounts of protein in each lane.

To determine whether cytotoxicity following H_2_O_2_ treatment was directly related to oxidative stress and whether AEMR could block it, the degree of ROS generation was investigated. Flow cytometry results by 2′7′-di-chlorodihydrofluorescein diacetate (DCF-DA) staining showed that ROS generation was significantly increased within 1 h in H_2_O_2_-treated C2C12 cells (Figure 4A,B). However, the frequency of DCF-positive cells increased by H_2_O_2_ were remarkably decreased by AEMR, and compound C significantly abolished the protective effect of AEMR. In addition, the effect of AEMR against H_2_O_2_-induced DNA damage was further evaluated by comet assay, a method to assess DNA damage induced by oxidative stress in individual cells [24]. As shown in Figure 4C,D, migration of damaged DNA fragments by electrophoresis was clearly observed in H_2_O_2_-treated cells, as compared to control cells. The expression level of phosphorylated nuclear histone H2A.X protein (γH2A.X), a DNA double-strand breaks marker [25], was also increased in cells treated with H_2_O_2_ alone. However, in the presence of AEMR, DNA tails were not generated, the expression of γH2A.X was maintained at control level, and the protective effect of AEMR against H_2_O_2_-induced DNA damage was attenuated by compound C (Figure 4). These findings are indicative that the AMPK-dependent protective effect of AEMR against DNA damage caused by H_2_O_2_ might be directly associated with the inhibition of ROS production.

### 2.5. AMPK Was Involved in the Protective Effect of AEMR against H_2_O_2_-Induced Mitochondrial Dysfunction in C2C12 Cells

**Figure 5:**
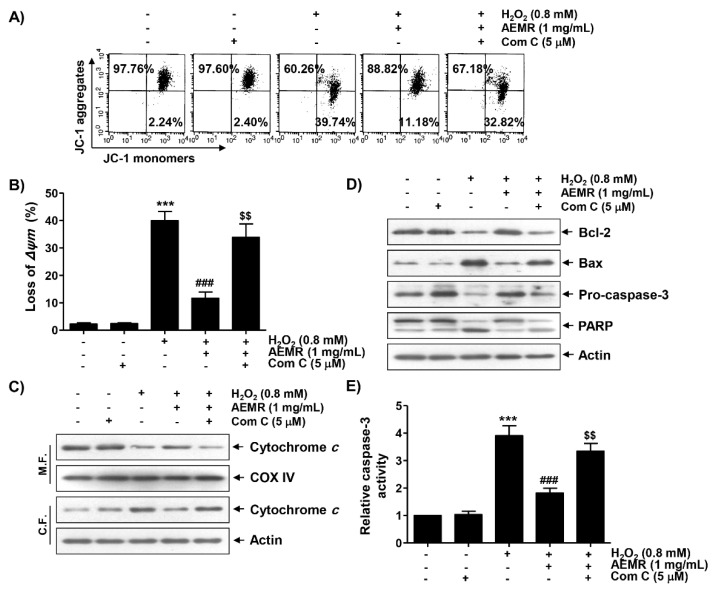
Effects of AMPK inhibitor on the protective effect of AEMR on MMP loss and changes in the expression of apoptosis regulators in H_2_O_2_-treated C2C12 cells. Cells were pretreated with 5 μM compound C with or without 1 mg/mL AEMR for 1 h, and then subjected to treatment with 0.8 mM H_2_O_2_ for 24 h. (**A**,**B**) MMP was assessed by flow cytometry after JC-1 staining. (**A**) Representative profiles of flow cytometry analysis are shown. (**B**) Ratios of JC-1 aggregates to monomers are presented as mean ± SD of triplicate independent experiments (*** *p* < 0.001 vs. control cells; ^###^ *p* < 0.001 vs. H_2_O_2_-treated cells; ^$$^ *p* < 0.01 vs. H_2_O_2_ and AEMR-treated cells). (**C**) Expression of cytochrome *c* using mitochondrial and cytoplasmic fractions was measured by western blot analysis. Cytochrome *c* oxidase subunit IV (COX IV) and actin were analyzed as internal controls for mitochondrial and cytosolic fractions, respectively. M.F., mitochondrial fraction; C.F., cytoplasmic fraction. (**D**) Using total proteins isolated from cells, the expression of indicated proteins was detected by western blot analysis. Actin was used to determine equal amount of protein loaded into each lane. (**E**) Activity of caspase-3 was measured using a colorimetric caspase-3 assay kit. Data are presented as mean ± SD in fold induction from three independent experiments (*** *p* < 0.001 vs. control cells; ^###^ *p* < 0.001 vs. H_2_O_2_-treated cells; $$ *p* < 0.01 vs. H_2_O_2_ and AEMR-treated cells).

To study whether AMPK was involved in the inhibitory effect of AEMR against H_2_O_2_-mediated mitochondrial dysfunction, the effect of AMPK inhibition on the protective effect of AEMR on H_2_O_2_-induced MMP loss was investigated. As shown in Figure 5A,B, the protective effect of AEMR on H_2_O_2_-induced MMP loss was significantly reduced when cells were pretreated with compound C. These results suggest the possibility that AEMR contributed to the preservation of mitochondrial function in H_2_O_2_-treated C2C12 cells through activation of AMPK. Therefore, we investigated the effect of AEMR on the expression of proteins involved in the induction of apoptosis related to mitochondrial dysfunction in H_2_O_2_-treated cells, and further evaluated whether AMPK was involved in this process. The results of immunoblotting using mitochondrial and cytoplasmic fractions showed that the expression of cytochrome *c* in the cytoplasm was increased under H_2_O_2_ stimulation conditions, while its expression in the mitochondria was decreased (Figure 5C). However, these changes were markedly blocked by AEMR. In addition, the expression of anti-apoptotic Bcl-2 protein was decreased by H_2_O_2_ treatment, whereas the expression of pro-apoptotic Bax protein was increased. Moreover, as the expression of the inactive form of caspase-3 was decreased by H_2_O_2_, its enzymatic activity was increased, which was correlated with the degradation of poly (ADP-ribose) polymerase (PARP), one of the substrate proteins cleaved by activated effector caspases such as caspase-3 [26,27] (Figure 5A,B). However, all these changes were blocked by AEMR pretreatment. At the same time, in the presence of compound C, the inhibitory effects of AEMR on cytochrome *c* leakage into the cytoplasm, changes in the expression of Bcl-2 family proteins, caspase-3 activation, and PARP cleavage were almost completely abolished (Figure 5).

### 2.6. The Protective Effect against H_2_O_2_-Induced Apoptosis by AEMR Was Achieved through Activation of AMPK in C2C12 Cells

**Figure 6:**
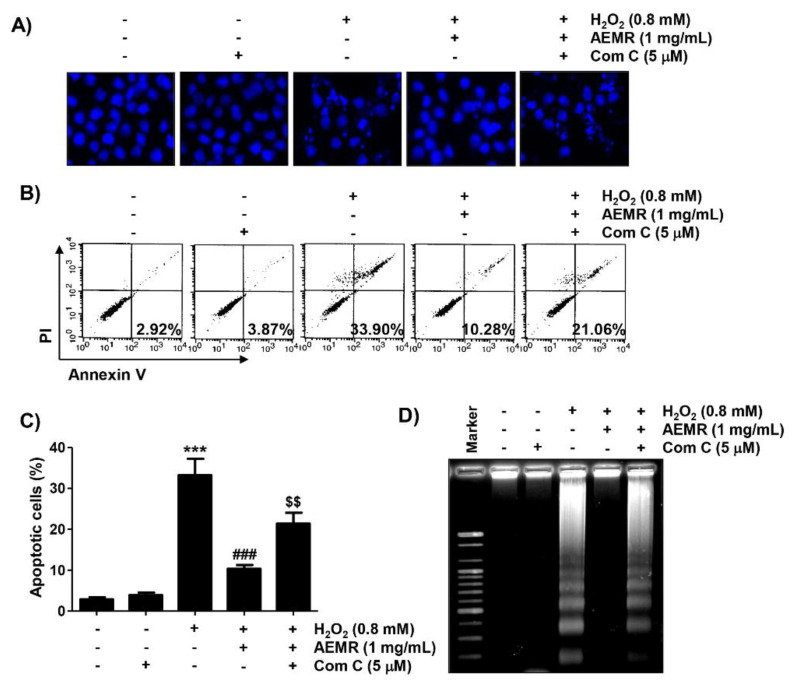
Effect of HO-1 inhibitor on AEMR-mediated protection of apoptosis by H_2_O_2_ in C2C12 cells. Cells were pretreated with 5 μM compound C with or without 1 mg/mL AEMR for 1 h, and then treated with 0.8 mM H_2_O_2_ for 24 h. (**A**) Induction of apoptosis was evaluated by measuring cells with condensed nuclei after 4′,6′-diamidino-2-phenylindole (DAPI) staining. Representative photomicrographs are shown. (**B**,**C**) Cells were stained with annexin V/PI and then analyzed by flow cytometry. (**B**) Percentages of apoptotic cells are shown in lower right panels. (**C**) Quantitative analysis of apoptotic cells in percentage, including early and late apoptotic cells. Each point is the mean ± SD of independent triplicate experiments (*** *p* < 0.001 vs. control cells; ^###^ *p* < 0.001 vs. H_2_O_2_-treated cells; ^$$^ *p* < 0.01 vs. H_2_O_2_ and AEMR-treated cells). (**D**) Fragmentation of genomic DNA isolated from cells was evaluated by agarose gel electrophoresis.

Finally, we investigated whether AMPK was involved in the survival enhancing effect of AEMR on H_2_O_2_-induced apoptotic cell death in C2C12 cells. Results of 4′,6′-diamidino-2-phenylindole (DAPI) staining revealed that the condensation of chromatin and the formation of apoptotic bodies were significantly increased in cells treated with H_2_O_2_ alone (Figure 6A). Flow cytometry analysis also showed that the frequency of annexin V-positive cells, representing apoptotic populations, was greatly increased by H_2_O_2_ treatment (Figure 6B,C). Consistent with these results, fragmentation of genomic DNA, a representative hallmark of apoptosis, was induced in H_2_O_2_-treated cells (Figure 6D). As expected, these indicators of apoptosis were significantly reduced in the presence of AEMR, suggesting that AEMR could effectively restore apoptosis caused by H_2_O_2_. However, the protective effect of AEMR against H_2_O_2_-induced apoptosis was abolished in the presence of compound C (Figure 6), supporting the hypothesis that AEMR protected against oxidative stress-induced damage by activating the AMPK signaling pathway in C2C12 cells.

### 2.7. The Protective Effect of AEMR against Dexamethasone-Induced Muscle Atrophy in Rats

**Figure 7:**
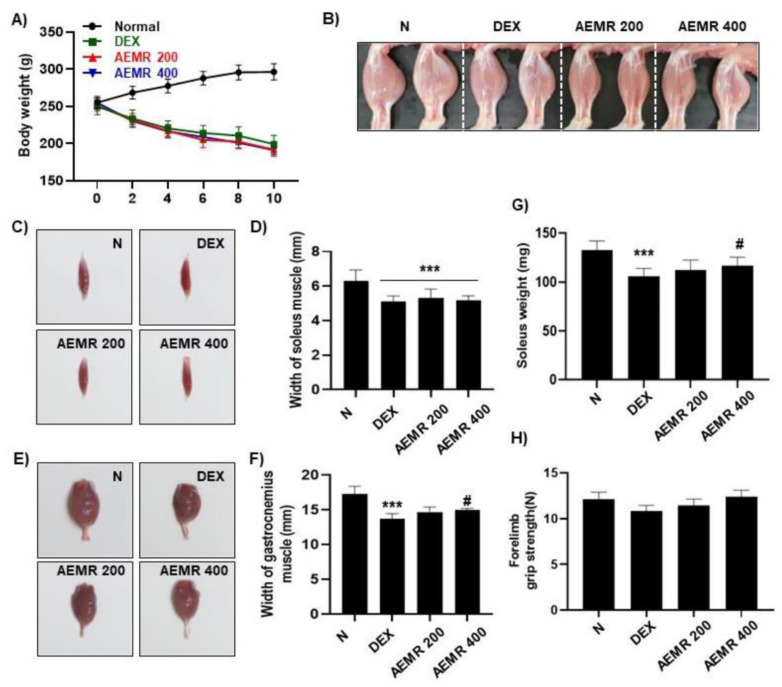
The protective effect of AEMR against dexamethasone (DEX)-induced muscle atrophy in Sprague-Dawley rats. (**A**) Effect of oral administration of AEMR on body weight. (**B**) Representative images of the hindlimbs of Sprague-Dawley rats. (**C**) Representative images of gastrocnemius muscle. (**D**,**G**) Width and weight of gastrocnemius muscle. (**E**) Representative images of soleus muscle. (**F**) Width of soleus muscle. (**H**) Forelimb grip strength in the study groups before sacrifice. Each values represent the average of 5 tests per animal. Data are shown as mean ± SD. *** *p* < 0.001 vs. Normal; ^#^ *p* < 0.05 vs. DEX.

To evaluate the effect of AEMR in vivo, we investigated the effect of oral administration of AEMR on dexamethasone (DEX)-induced muscle atrophy in Sprague-Dawley rats. As shown in Figure 7A, body weight was significantly decreased following DEX treatment, but DEX-induced body weight loss was not recovered by oral administration of AEMR. Although AEMR treatment did not improve decreasing soleus muscle thickness by DEX treatment, soleus weight was markedly increased in the AEMR 400 group (Figure 7B–D,G). Furthermore, oral application of 400 mg/kg AEMR significantly enhanced the width of the gastrocnemius muscle compared with the DEX-treated group (Figure 7E,F). However, no significant difference was observed in the forelimb grip strength among the groups (Figure 7H). This result showed that oral administration of AEMR alleviated gastrocnemius and soleus muscle loss in DEX-induced muscle atrophy, which supported that AEMR might be a promising therapeutic candidate for treating muscle atrophy.

## 3. Discussion

C2C12 mouse myoblasts, precursor cells in adult mouse skeletal muscle tissue, have been widely used in an in vitro model against various oxidative stresses ever since it was found that oxidative stress using H_2_O_2_ could damage myoblasts to mimic exercise-induced changes in skeletal muscle [28,29]. In this study, we investigated the effect of AEMR on H_2_O_2_-induced cytotoxicity in C2C12 myoblasts, and found that AEMR significantly inhibited H_2_O_2_-induced DNA damage and apoptosis, which was associated with suppression of ROS production. We also found that the antioxidant activity of AEMR was achieved through activation of the AMPK signaling pathway. 

Oxidative stress can cause damage to various intracellular organelles and macromolecules, so failure to restore sustained oxidative stress eventually induces apoptosis, resulting in tissue and organ injury [10,30]. In particular, in skeletal muscle susceptible to oxidative stress, mitochondrial impairment serves as an initiation signal for activation of the endogenous apoptosis pathway [3,5,31,32]. In addition, oxidatively damaged DNA induces genetic mutations, and disrupts the homeostasis of muscle function, which not only causes muscle damage, but also causes the failure of inducing differentiation of myoblasts into muscle cells [8,33]. However, a variety of intracellular signaling molecules and enzymes are involved in the maintenance of redox homeostasis in the cells for defense against oxidative stress. Among them, AMPK plays an important role in redox homeostasis as well as energy metabolism [34,35]. AMPK also participates as a key regulator in the induction of cell growth, differentiation and proliferation, autophagy, and apoptosis, [36,37]. In addition, AMPK has been found to maintain the biogenesis of mitochondria through controlling ROS generation in cells with active energy metabolism, including skeletal muscle [4,23]. Mitochondria, which are completely responsible for energy production in the cells, are vulnerable to oxidative stress, and several studies have shown that the antioxidant activity of extracts of *Morus alba* or its components was related to the defense of mitochondrial damage caused by various stimuli [38,39]. Mitochondrial membrane depolarization is a typical feature that appears when mitochondria are damaged, and the loss of MMP is an indicator that mitochondrial integrity is disrupted [7,40]. 

In this study, AEMR exhibited a significant protective effect against H_2_O_2_-induced cytotoxicity, which was also confirmed through the reduction of irregular cell morphology due to H_2_O_2_ treatment (Figure 1), indicating that AEMR was able to protect against H_2_O_2_-induced cytotoxicity in C2C12 cells. In addition, H_2_O_2_-induced loss of MMP was significantly restored in the presence of AEMR in a concentration-dependent manner, suggesting that AEMR inhibited mitochondrial damage caused by oxidative stress. Therefore, we investigated whether AEMR could induce activation of AMPK, and found that AEMR activated AMPK in H_2_O_2_-stimulating conditions (Figure 2). However, AEMR-mediated reversion of H_2_O_2_-induced cytotoxicity was abolished by pretreatment with compound C, an inhibitor of AMPK (Figure 3), suggesting that activation of AMPK might be involved in the protective effect of AEMR against oxidative stress-mediated cytotoxicity in C2C12 cells. We also found that AEMR had the ability to ameliorate H_2_O_2_-induced DNA damage while blocking ROS production. We also found that AEMR had the ability to ameliorate H_2_O_2_-induced DNA damage by blocking H_2_O_2_-induced ROS generation. However, compound C abrogated the protective effect of AEMR against H_2_O_2_-induced ROS generation and DNA damage (Figure 4). These results revealed that activation of AMPK was involved in the protective effect of AEMR against oxidative stress-mediated DNA damage in C2C12 myoblasts.

**Figure 8:**
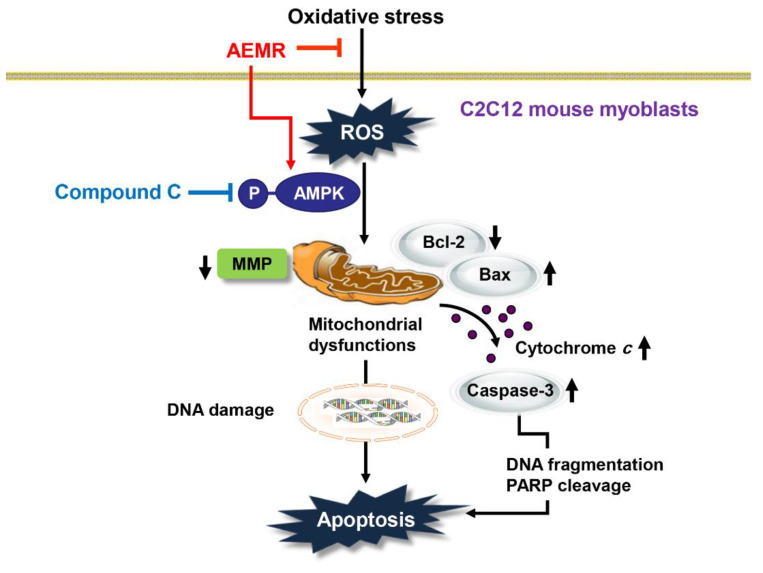
Schematic summarizing the protective effects of AEMR against oxidative stress-induced DNA damage and apoptosis in C2C12 myoblasts via AMPK activation.

It is well known that the induction of apoptosis in C2C12 cells by H_2_O_2_ is due to the release of apoptotic proteins, such as cytochrome *c*, into the cytoplasm by disruption of mitochondrial membrane stability [29,41]. This is a typical process of the initiation of the intrinsic pathway among the apoptosis pathways, and is tightly regulated by members of the Bcl-2 family, which consist of anti-apoptotic and pro-apoptotic proteins [8,26]. Bcl-2 family proteins regulate the permeability of the mitochondrial outer membrane, thereby regulating the release of cytochrome *c* from the mitochondria to the cytoplasm [26,27]. Consequently, we found that cytochrome *c* expression was predominantly expressed in the cytoplasm in H_2_O_2_-treated cells, but its expression was counteracted by pretreatment with AEMR, suggesting that mitochondrial integrity was maintained in the presence of AEMR. In addition, increased expression of pro-apoptotic protein Bax and decreased expression of anti-apoptotic protein Bcl-2 by H_2_O_2_ were reversed by AEMR pretreatment. This was linked to inhibiting the degradation of substrate proteins, such as PARP, by blocking the activity of caspase-3 (Figure 5). Therefore, it is presumed that the increase in the relative expression of Bcl-2 to Bax by AEMR in C2C12 cells played a decisive role in blocking H_2_O_2_-induced apoptosis (Figure 6). However, the inhibitory effects of AEMR against H_2_O_2_-mediated apoptosis events, including mitochondrial dysfunction, were completely abolished by the AMPK inhibitor, suggesting that AEMR conferred protection against oxidative stress-induced cellular damage through AMPK activation. These finding revealed AEMR may be an effective therapeutic antioxidant to prevent apoptosis caused by inhibiting ROS generation, which is the cause of mitochondria-mediated apoptosis pathway. Furthermore, our data demonstrated that AEMR-mediated activation of AMPK contributed to the inhibition of H_2_O_2_-induced mitochondrial dysfunction and apoptosis. Therefore, we speculate that the protective effect of AEMR against oxidative stress-induced C2C12 myoblast damage was at least due to suppression of mitochondrial damage through AMPK activation (Figure 8). However, since the AMPK inhibitor hampered this protective effect, we propose that activation of AMPK by AEMR may be at least involved in protection from oxidative stress-induced C2C12 myoblast damage. Nevertheless, we cannot exclude the involvement of other signaling pathways, including upstream and downstream molecules of AMPK, that may contribute to the regulation of mitochondrial homeostasis and ROS generation in myoblasts.

To date, chemical perspective studies have identified that approximately 20 phytochemicals, including a number of flavonoids, caffeoylquinic acid, maclurin, etc., with antioxidant potential have been successfully isolated from *M. alba* [42,43,44,45]. In 2017, Jiang et al. [43] successfully isolated seven phytophenols, including five flavonoids (morin, rutin, astragalin, isoquercitrin and luteolin) and two non-flavonoids (chlorogenic acid and maclurin), from *Mori Fructus* and *Mori Ramulus*. More recently, Chen et al. [44] developed the HPLC method for identification of effective substance and discrimination of *Cortex Mori*, *Ramulus Mori*, *Folium Mori* and *Fructus Mori* using 13 marker compounds. They suggested the reference compounds should be the following: neochlorogenic acid, mulberroside A, chlorogenic acid, cryptochlorogenic acid, rutin, isoquercitrin, astragalin, morin, quercetin, kaempferol, sanggenon C, kuwanon G, and morusin. They found that quercetin, morin, kuwanon G, sanggenon C, morusin, mulberroside A, and rutin were chemically distinct among the various medicinal parts of *M. alba*. In addition, our previous study showed that AEMR contained rutin hydrate and astragalin, and the quantity of rutin hydrate was higher than astragalin [45]. Although we couldn’t have verified all active components of AEMR in the present study, we considered that this finding could support the results of previous studies demonstrating the bioactive activity of *Ramulus Mori*.

Muscle atrophy, which is triggered by several pathological conditions, such as cachexia, starvation, diabetes, metabolic acidosis, sepsis, and chronic kidney disease, while circulating glucocorticoid levels are elevated in these diseases, contributes to the reduction in muscle strength and muscle mass [46]. There have been reported that high doses or long-term use of synthetic glucocorticoid, such as DEX, induce muscle atrophy through physiological muscle changes in rodents [47,48]. DEX can induce catabolic muscle atrophy accompanied by characteristic histopathological changes, including loss of cell organelles, and decrease of protein content and muscle fiber diameter [49]. In this regard, numerous studies have suggested the beneficial effect of traditional medicinal herbs and their bioactive compounds on DEX-induced muscle atrophy models [48,50,51]. Kweon et al. [48] reported that the ethanol extract of Ashitaba, *Angelica keiskei* Koidzumi, and its active principle 4-hydroxyderricin can enhance muscle strength in DEX-induced muscle atrophied rats. Additionally, Kim et al. [50] demonstrated that the ethanol extracts of *Fructus Schisandrae*, the dried fruit of *Schizandra chinensis* Baillon, has a favorable ameliorating effect on muscle atrophy induced by DEX, by exerting anti-inflammatory and antioxidant effects on muscle fibers. More recently, there have been reported *schisandrin A*, a component extracted from the fruits of *S. chinensis*, reduces protein degradation, and increases protein synthesis in the muscle, contributing to the amelioration of DEX-induced muscle atrophy [51]. In the present study, we also verified that oral administration of AEMR alleviated gastrocnemius and soleus muscle loss in DEX-induced muscle atrophied rats. Although verification of their effectiveness for the physiologically active ingredients of AEMR should be conducted, our findings support that AEMR might be a promising therapeutic candidate for treating oxidative stress-mediated myoblast injury and muscle atrophy.

## 4. Materials and Methods

### 4.1. AEMR Preparation

Mori Ramulus was kindly obtained from the Bohyeonsan Clean Herb Farming Association (Yeongcheon, Korea), and the voucher specimens were deposited in the Herbarium, Department of Biochemistry, Dong-Eui University College of Korean Medicine (Busan, Korea). The extraction procedure for AEMR was applied as follows: 500 g of Mori Ramulus was ground and extracted with 2 L of boiled distilled water for 1 h using a reflux system (Kyungseo E&P, Incheon, Korea). After cooling, the decoctions were filtered using Whatman paper, and freeze-dried to a fine powder using a freeze dryer (IlShinBioBase, Dongducheon, Korea). The samples (AEMR) were stored in a −80 °C freezer until being used for experiments.

### 4.2. Cell Culture and AEMR Treatment

C2C12 murine myoblasts were purchased from the American Type Culture Collection (Manassas, VA, USA), and maintained in Dulbecco’s modified Eagle’s medium (DMEM; WelGENE Inc., Gyungsan, Korea), supplemented with 1% antibiotics (penicillin/streptomycin, WelGENE Inc., Gyungsan, Korea) and 10% fetal bovine serum (WelGENE Inc., Gyungsan, Korea) in a humidified 5% CO_2_ incubator at 37 °C. AEMR and H_2_O_2_ (Sigma-Aldrich Chemical Co., St. Louis, MO, USA) were dissolved in Milli-Q Water to prepare stock solutions, followed by dilution with DMEM to appropriate concentrations.

### 4.3. Cell Viability 

Cell viability was measured using an MTT assay as previously described [41]. Briefly, C2C12 cells were treated with appropriate concentrations of AEMR, H_2_O_2_, or compound C for 24 h, or pretreated with AEMR in the presence or absence of compound C for 1 h, followed by H_2_O_2_ treatment for 24 h. After the treatments, the medium was removed, and the cells were incubated with MTT solution (0.5 mg/mL, Sigma-Aldrich Chemical Co., St. Louis, MO, USA) at 37 °C for 2 h. Subsequently, the formed formazan was dissolved with dimethyl sulfoxide (DMSO, Sigma-Aldrich Chemical Co., St. Louis, MO, USA) The absorbance was then detected at 540 nm using an enzyme-linked immunosorbent assay (ELISA) reader (Molecular Devices, Sunnyvale, CA, USA). Results are expressed as the survival rate of cells relative to that of the control group. The cell morphology was observed under an inverted contrast phase microscope (Carl Zeiss, Oberkochens, Germany).

### 4.4. Analysis of MMP

To analyze MMP, JC-1 staining was performed after treatment of the cells with the indicated concentrations of AEMR in the presence or absence of H_2_O_2_ or compound C for the indicated durations. Subsequently, the collected cells were washed with phosphate-buffered saline (PBS), and stained with 10 μM JC-1 solution (Sigma-Aldrich Chemical Co., St. Louis, MO, USA) in the dark for 20 min at RT, according to the manufacturer’s procedure. After removing the supernatant, the cells were washed again with PBS, and analyzed using a flow cytometer (Becton Dickinson, San Jose, CA, USA) to measure MMP. Results are expressed as a ratio of JC-1 aggregates, indicating the extent of mitochondrial depolarization to form JC-1 monomers [52]. 

### 4.5. Protein Isolation and Western Blot Analysis 

For the expression analysis of target proteins by immunoblotting, whole-cell extracts from the treated cells were extracted according to published method [41]. Cytoplasmic and mitochondrial proteins for cytochrome *c* expression analysis were extracted using a mitochondrial fractionation kit (Active Motif, Inc., Carlsbad, CA, USA) in accordance with the manufacturer’s instructions. After quantifying isolated proteins, the same amount of protein was mixed with Laemmli sample buffer (Bio-Rad Lab., Hercules, CA, USA), and then separated by sodium-dodecyl sulfate (SDS)-polyacrylamide gel electrophoresis. After electrophoresis, proteins were electrotransferred onto PVDF membranes (Millipore, Bedford, MA, USA)) for 1 h at RT. The membranes were incubated with 5% skim milk for 1 h at room temperature (RT) to block non-specific proteins, and then probed with primary antibodies (Santa Cruz Biotechnology, Inc., Santa Cruz, CA, USA, Abcam, Inc., Cambridge, UK, and Cell Signaling Technology, Danvers, MA, USA) overnight at 4 °C. After washing with PBS-T (PBS with Tween 20), membranes were blotted with horseradish peroxidase-conjugated secondary antibodies (Santa Cruz Biotechnology, Inc. Santa Cruz Biotechnology, Inc., Santa Cruz, CA, USA) suitable for primary antibodies for 1 h at RT, and then exposed to enhanced chemiluminescence solution (Amersham Biosciences, Westborough, MA, USA) to visualize the protein of interest according to the manufacturer’s instructions. 

### 4.6. Analysis of ROS Content

To detect the level of ROS generated in cells, DCF-DA staining was performed. To this end, cells were treated with the indicated concentrations of H_2_O_2_ or compound C (Sigma-Aldrich Chemical Co., St. Louis, MO, USA) for 1 h, or pretreated with AEMR and compound C for 1 h followed by treatment with H_2_O_2_ for an additional 1 h. Subsequently, the medium was replaced with a solution of 10 μM DCF-DA (Sigma-Aldrich Chemical Co., St. Louis, MO, USA) in medium, and incubated for 20 min in the dark. After staining, the levels of ROS production were evaluated using a flow cytometer, at excitation/emission wavelengths of 488/525 nm, according to the method described previously [53]. 

### 4.7. Alkaline Comet Assay (Single Cell Gel Electrophoresis)

The effect of AEMR on DNA damage caused by H_2_O_2_ was determined using a CometAssay^®^ kit (Trevigen Inc., Gaithersburg, MD, USA)) per manufacturer’s instructions [54]. Briefly, cells treated with H_2_O_2_ in the absence or presence of AEMR and compound C were trypsinized and suspended in ice-cold PBS. The cells were then mixed with low melting agarose, and immediately spread onto slide glasses. After agarose was solidified, slides were submerged with a lysis solution supplied in the kit. Electrophoresis was then performed at 25 °C for 30 min at 25 V. After electrophoresis, the cells were stained with ethidium bromide (EtBr, Sigma-Aldrich Chemical Co., St. Louis, MO, USA), and nuclei images were captured under a fluorescence microscope (Carl Zeiss, Oberkochens, Germany).

### 4.8. Caspase-3 Activity Assay

Activity of caspase-3 was measured using a caspase-3 ELISA kit (R&D Systems, Inc., Minneapolis, MN, USA). Briefly, the cells treated with H_2_O_2_ in the absence or presence of AEMR and compound C were trypsinized and suspended in ice-cold PBS. After lysing cells to be measured, the supernatant was reacted with a reaction buffer per the recommendation of the manufacturer. The optical density of the reaction mixture of each sample after the reaction was measured at 405 nm using an ELISA reader, and expressed as a relative value [52]. 

### 4.9. DAPI Staining

The nuclear morphological changes of the cells were observed using a DAPI fluorescence staining, as previously described [41]. Following treatment, the cells were washed with PBS and fixed with 4% paraformaldehyde (Sigma-Aldrich Chemical Co., St. Louis, MO, USA) for 20 min at RT. After permeabilization, the cells were stained with a DAPI solution (Sigma-Aldrich Chemical Co., St. Louis, MO, USA) at 37 °C for 20 min in the dark. Thereafter, the cells were washed again with PBS, and photographed under a fluorescence microscope.

### 4.10. Annexin V/PI Staining Assay

Annexin V/PI double staining was used to measure the proportion of apoptotic cells. To this end, cells treated with H_2_O_2_ in the absence or presence of AEMR and compound C were collected, washed with PBS twice, and resuspended in binding buffer provided in the kit. Subsequently, the cells were stained using annexin V/PI solution (Becton Dickinson, San Jose, CA, USA) for 20 min at RT in the dark, according to the manufacturer’s protocol. Finally, the cells were immediately subjected to flow cytometry to quantify annexin V-positive cells as apoptosis-induced cells, following published procedures [55].

### 4.11. DNA Fragmentation Assay

To confirm the presence of internucleosomal DNA cleavage, DNA gel electrophoresis was performed after treatment of cells with H_2_O_2_ in the absence or presence of AEMR and compound C. Briefly, the collected cells were washed with PBS and resuspended in lysis buffer on ice for 30 min, as previously described [56]. The cells were incubated with 1 μg/mL RNase A (Sigma-Aldrich Chemical Co., St. Louis, MO, USA) for 2 h at 37 °C, and then genomic DNA was extracted from the supernatant with phenol/chloroform/isoamyl alcohol (Sigma-Aldrich Chemical Co., St. Louis, MO, USA). After precipitation with ethanol, the DNA was resolved by electrophoresis on 1.5% agarose gel at 70 V. The gel was stained with 0.1 µg/mL EtBr, and the DNA ladders were subsequently visualized with a UV transilluminator (Vilber, Collégien, France) at the Core Facility Center for Tissue Regeneration, Dong-Eui University (Busan, Korea).

### 4.12. Animal Experiments

All animal studies were approved by the Institutional Animal and Use Committee of Dong-Eui University (Approved No. R2019-003), and were performed in accordance with the guidelines for animal experimentation issued by Dong-Eui University. Six-week-old Sprague Dawley rats were obtained from Samtako (Osan, Korea). Rats were maintained under controlled environmental conditions under a 12 h/12 h light/dark cycle, and were allowed ad libitum access to water and a standard laboratory diet. After accumulation for one week, rats were randomly divided into four groups: (i) the intact vehicle control group, (ii) DEX control group, (iii) DEX and AEMR 200 mg/kg administered group, and (iv) DEX and AEMR 400 mg/kg administered group (8 animals per group), as previously described [57]. To induce muscle atrophy, DEX (600 μg/kg body mass) was intraperitoneally injected daily for 10 days. Two different concentrations of AEMR (200 and 400 mg/kg body mass) were orally administered once daily for the same period. After ten days, rats were tested for forelimb grip strength using a digital strength meter (Columbus Instruments, Columbus, OH, USA). In brief, forelimb strength testing involved placing the animals’ two forelimb paws on a metal grid adjoined to an electronic force transducer. The animal was then horizontally pulled away from the grid, and force readings were provided upon the point at which the animal could no longer maintain grip contact. This test was repeated three times per animal, and the average of all three trails was used for statistical analyses [58]. After measurement, all rats were sacrificed by decapitation, soleus and gastrocnemius muscles were quickly removed, and the weight was immediately measured as previously described [59].

### 4.13. Statistical Analysis

One-way ANOVA with Tukey’s post hoc comparisons was used for multiple comparisons. Analysis was performed using Prism 6.0 software (GraphPad Software Inc., La Jolla, CA, USA). Data were expressed as mean ± standard deviation (SD). *p* values < 0.05 were considered statistically significant.

## 5. Conclusions

In summary, AEMR significantly blocked H_2_O_2_-induced DNA damage and apoptotic cell death, which was correlated with its ability to block ROS production and mitochondrial dysfunction. We also found that the apoptosis-blocking effect of AEMR was associated with the inhibition of mitochondria-mediated intrinsic apoptosis pathway through regulation of Bcl-2 family proteins, and inhibition of cytoplasmic release of cytochrome *c*. Furthermore, we verified that oral administration of AEMR alleviated gastrocnemius and soleus muscle loss in DEX-induced muscle atrophied rats. Our findings support that AEMR might be a promising therapeutic candidate for treating muscle atrophy through suppression of oxidative stress-mediated myoblast injury.

## Data Availability

Data presented in this study are available on request from the corresponding author.
